# Retraction: l-Arginine Uptake by Cationic Amino Acid Transporter Promotes Intra-Macrophage Survival of *Leishmania donovani* by Enhancing Arginase-Mediated Polyamine Synthesis

**DOI:** 10.3389/fimmu.2024.1474938

**Published:** 2024-08-14

**Authors:** Frontiers Editorial Office

Following publication, concerns were raised regarding the integrity of the images in the published figures.

The authors failed to provide a satisfactory explanation during the investigation, which was conducted in accordance with Frontiers’ policies. As a result, the data and conclusions of the article have been deemed unreliable and the article has been retracted. The authors do not agree to this retraction.

